# Applicability of intraoral scans for presurgical orthopedic treatment of cleft lip and palate in infants: a prospective non-randomized comparative study

**DOI:** 10.1007/s00784-025-06444-9

**Published:** 2025-07-09

**Authors:** Christiane Keil, Michaela Buckova, Sheila Keil, Louis Hartmann, Wilhelm Schlupp, Thomas Zerjatke, Theodosia Bartzela, Günter Lauer

**Affiliations:** 1 Department of Orthodontics, University Hospital Carl Gustav Carus, Technische Universität Dresden, Fetscherstr. 74, Haus 28, D-01307 Dresden, Germany; 2 Department of Oral Maxillofacial Surgery, University Hospital Carl Gustav Carus, Technische Universität Dresden, Dresden, Germany; 3 Institute for Medical Informatics and Biometry (IMB), Faculty of Medicine, Technische Universität Dresden, Dresden, Germany

**Keywords:** Cleft lip, Cleft palate, Orthopedic proceures, Dental models

## Abstract

**Objectives:**

This in vivo study evaluated the reliability of digital impressions from intraoral scans compared to digital scans of plaster casts of the edentulous maxilla of infants with cleft lip and palate (CLP).

**Materials and methods:**

To compare and evaluate the dimensional accuracy of digital and conventional impressions, 52 infants with CLP were selected. Conventional plaster and digital impressions were taken from each patient before surgical closure of the lips and/or palate. Plaster casts were digitized using the intraoral scanner. Eight linear distances were measured and 3D superimpositions were performed. Pearson’s correlation, mean difference and root mean square were calculated to compare the two impression methods.

**Results:**

Regardless of the form of CLP, the IOS and the plaster casts correlated very well. The measured linear dimensions of the IOS were greater than those of the digitized plaster casts. Although the linear measurements showed differences of up to 0.89 mm, superimpositions showed that the two impression methods did not differ in a clinically relevant way.

**Conclusions:**

No clinically relevant difference in accuracy was found between IOS and the plaster casts. When investigating the accuracy, 3D analysis should always be preferred to 2D analysis.

**Clinical relevance:**

This study confirms that IOS is a reliable and clinical valuable alternative for infants with CLP. IOS eliminates risks associated with traditional methods, providing a safer and more efficient workflow for PSIO. Despite minor differences in dimensional accuracy, these variations are clinically negligible and do not impact treatment planning or appliance fabrication.

## Introduction

Cleft lip and palate (CLP) is one of the most common craniofacial malformations. The incidence among newborns is between 1:500 and 1:2000 and varies from region to region [1, 2]. In Germany, about 50% of cleft malformations are cleft lip and palate (CLP, primary and secondary palate), followed by isolated cleft palate (CP) or cleft lip (CL) and cleft lip and palate (CLP, primary palate only) [3]. As early as 1950, presurgical infant orthopedics (PSIO) was used for the first time in cleft patients [4]. Even though the effect of PSIO treatment on the maxillary arch and the alveolar processes has been controversial [5], PSIO remains the treatment of choice for most the cleft centres [6]. An intraoral maxillary impression is required to perform PSIO. Due to the risk of aspiration of the impression material and decreasing oxygen saturation, conventional impressions pose a risk for infants [7, 8]. Furthermore, residual impression material can cause inflammation in the cleft [9]. In contrast, digital impression techniques eliminate the risk associated with impression materials and could, therefore, be a safer alternative.

Since their introduction in 1990, intraoral scanners have quickly found their way into clinical practice and have been used more and more frequently ever since [10–12]. As early as 2014, it was claimed that digital impressions taken with an intraoral scanner would replace analog impressions [13], but this has not yet been the case. It must first be demonstrated that impressions taken with the intraoral scan achieve the same level of accuracy as with alginate.

Numerous efforts have been made to investigate the diagnostic accuracy and equivalence of digital models compared to plaster models from alginate impressions. It has already been demonstrated that the dimensional differences between intraoral scans and conventional impression methods are clinically irrelevant in mixed dentition. The mean difference between the two impression methods was 0.022 ± 0.027 mm [14]. Aswani’s work also showed comparable and promising accuracy of the intraoral scans. Furthermore, it became clear that accuracy depends on the type of intraoral scanner and software used [15]. Furthermore, the systematic review by Kong et al. revealed a 3D deviation between digital and alginate full-arch impressions of only 0.09 mm, while Ahlholm and coworkers reported that the conventional method still provides the best results for full-arch impressions [16, 17]. The systematic review of the quantitative 3D accuracy of digital and conventional implant impressions in partially and fully edentulous patients showed that digital impressions were comparable to traditional impressions under in vitro conditions [18]. At present, there are only a few publications on the use of digital impressions in patients with CLP [19–24]. Furthermore, no studies have yet assessed the accuracy of intraoral scans compared to conventional alginate impressions in this group of patients. Therefore, the aim of this in vivo study was to compare the digital impressions from intraoral scans with digital scans of plaster casts in edentulous maxilla of infants with CLP in order to identify any dimensional differences (primary outcome) between the two impression methods. Two methods were used to compare the accuracy of digital and conventional impressions: linear measurement and 3D superimposition. The focus was not on a specific CLP subtype but on assessing the accuracy of different impression methods for the fabrication of PSIOs. Since PSIOs can be used for unilateral CLAP (UCLAP), bilateral CLAP (BCLAP), and CP, children with various CLP subtypes were included in this study. The following null hypothesis was accepted: There is no clinically relevant dimensional difference between the intraoral scans and the digitized plaster models in infants with CLP.

## Materials and methods

### Study design

The non-randomized comparative study was performed by the principles outlined in the Declaration of Helsinki in 2013 and approved by the Ethics Committee of the Technische Universität Dresden (SR + BO-EK-43012020) at the Department of Oral Maxillofacial Surgery, University Hospital Carl Gustav Carus of Dresden, Germany. The written informed consent was obtained from each legal guardian of participating patients with CLP prior to the start of the study.

Intraoral scans and alginate impressions were obtained from 52 Caucasian infants before the cleft lip repair and palatoplasty for general comparison. Intraoral scans and alginate impressions were available for 8 infants during both lip and palate closure. Cleft lip repair was performed on children between 4 and 6 months (mean age of 22.3 ± 7.7 weeks) and palate surgery between 9 and 12 months of age (mean age of 39.8 ± 5.9 weeks). Finally, a total of 60 intraoral scans as well as plaster casts from 52 patients with different cleft types were analyzed in this study (Table 1).

**Table 1:** Baseline characteristics of the included patients with CLP; cl = cleft lip; cp = cleft palate; uclap = unilateral cleft, alveolus, and palate; bclap = bilateral cleft, alveolus, and palate

Number of Scans / patients	Gender distribution male/female	Cleft type (number of scans)			
		CL	UCLAP	BCLAP	CP
60 / 52	30 / 22	6	25	13	16

### Interventions

Prior to the actual surgical procedure, all impressions were taken under general anaesthesia. The alginate jaw impressions (Tetrachrom^®^, Kaniedenta, Herford, Germany) and the production of plaster models were routinely performed by the staff of the Department of Maxillofacial Surgery and the dental laboratory. The plaster models were digitized using the *Trios*^®^*4 Wireless* intraoral scanner (3Shape, Copenhagen, Denmark).

The infants with CLP were also scanned intraorally with the above-mentioned scanner using a standardized scanning protocol. First, the intraoral scanner was inserted as deeply as possible into the center of the child’s mouth. The scan head was then swung to the right to capture the tuberosity. The alveolar ridge was captured by withdrawing the scanner. The scan was then continued on the left side, starting with the tuberosity. Finally, the lip frenulum and upper lip were scanned. The digital dental impressions were saved and exported in Standard Tessellation Language (STL) file format for further analysis using OnyxCeph3™ software (image-instruments, Chemnitz, Germany).

**Fig. 1:**
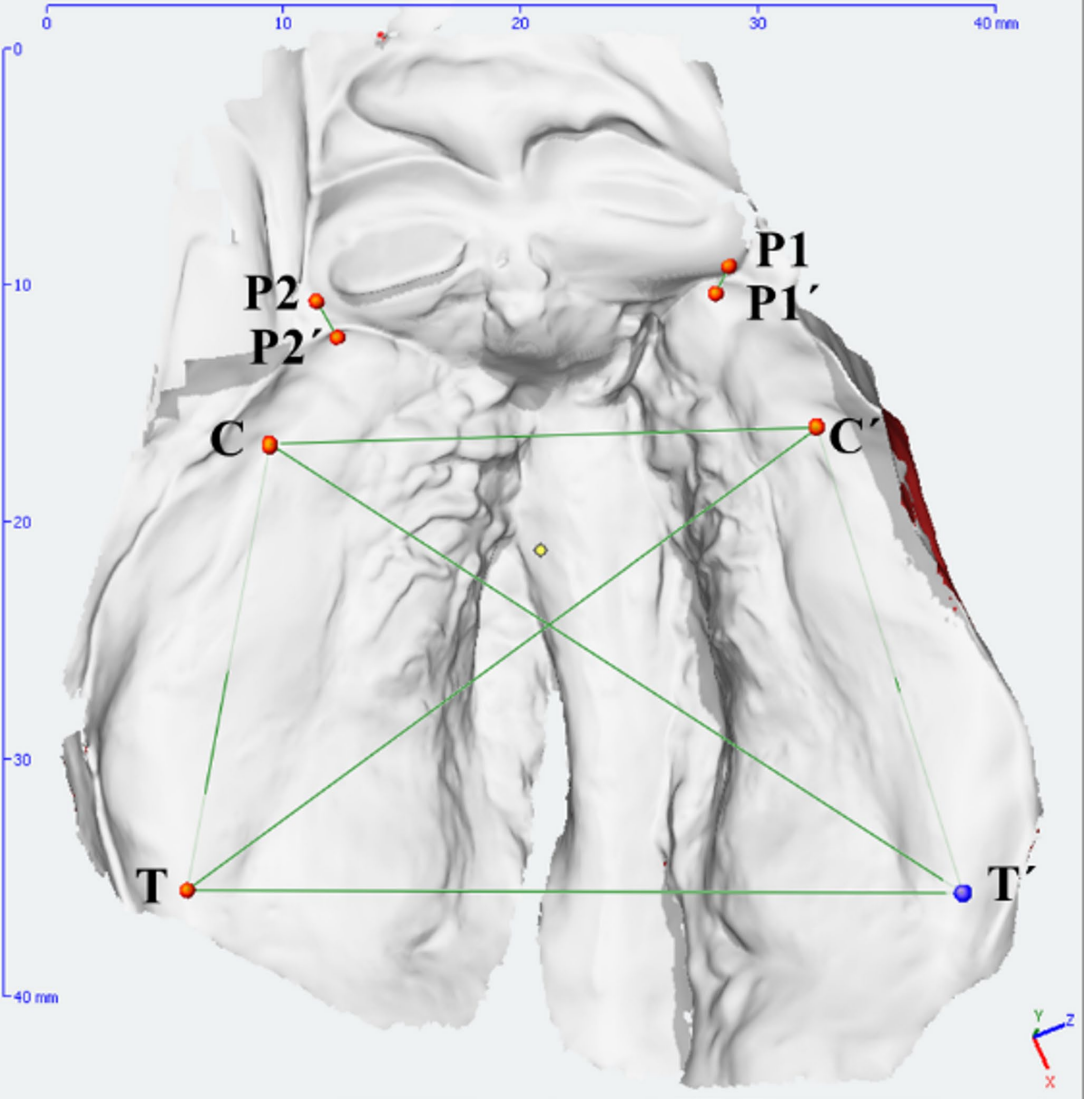
Anatomic points and linear measurements manually marked are illustrated on an intraoral scan of the bilateral cleft lip, alveolus, and palate (BCLAP)

Seven linear measurements were taken to compare the impression methods (Tables 2 and 3; Fig. 1). Three independent measurements were taken by two independent examiners. A time interval of at least one week was maintained between repeated measurements of the same model within the measuring procedure.

**Table 2:** Defined landmarks and reference points

Landmarks	Reference points	Definitions
T	Tuberosity point on the right	Junction of the alveolar ridge crest with the tuberosity outline
T´	Tuberosity point on the left	
P2´	Cleft edge point on the right	Cleft edges of the ridges
P1´	Cleft edge point on the left	
P2	Cleft edge point on the right	
P1	Cleft edge point on the left	
C	First canine point on the right	Junction of the lateral sulcus with the alveolar ridge crest (it refers to the dental papilla between canine and first primary molar)
C´	First canine point on the left	

**Table 3:** Outcome parameter definitions

Outcome parameters	Definition
**C-T**	Left lateral alveolar segment length
**C´-T´**	Right lateral alveolar segment length
**P1-P1´**	Left anterior cleft width
**P2-P2´**	Right anterior cleft width
**C-C´**	Intercanine distance– reciprocal canine points on the alveolar ridge
**T-T´**	Intertuberosity distance–reciprocal tuberosity points
**T-C´/ T´-C**	Diagonal distance between the tuberosity and canine points (for determining 2D-distortions

### Superimposition

The superimposition of the intraoral scans with the digital plaster models was carried out using the stl. files and current version of the free software Cloud Compare 2.14. alpha (https://cloudcompare-org.danielgm.net/release/) for 10 patients in the Cl/CP and UCLAP group. In the BCLAP group, a total of 9 superimpositions were evaluated. In the first step, the superimpositions were superimposed using defined landmarks (tuber point, canine tooth and first incisor). In the second step, the best fit algorithm was used with an accuracy of Δ 10^− 8^. The surface deviation data was determined by the software to document the accuracy between the intraoral scans and the plaster casts by calculating the root mean square (RMS) deviation. The RMS is typically used as a criterion to measure the similarity of two data files after optimal superimposition. A large error resulted in a higher calculated RMS, while lower RMS values correspond to higher accuracy. For superimposition, the stl files of the intraoral scans and the digital plaster casts were aligned and rearranged so that they were optimally aligned to each other. Finally, the distances between the both surfaces were converted to RMS as described by Jeong et al. [25].

### Statistical analysis

Wolfram Mathematica 14.1 (Wolfram Research, Inc. Champaign, United States) was used for data analysis and visualization. Measurements on digital models from plaster casts were defined as the gold standard, and the results of these measurements were compared with measurements on digital models from intraoral scanning. Pearson’s correlation and mean differences (Δ) were calculated to compare the intraoral scan with the plaster cast. A deviation of > 0.3 mm was identified as clinically relevant for colour maps [26, 27]. No clinically relevant deviation was determined for linear measurements, as all information available in the literature are not transferable to CLP infants.

To estimate the intra-observer variation of the measurement procedure, the standard deviation was calculated for three repeated measurements, and then averaged across all positions and patients. To test for the superimpositions whether the mean RMS value deviates from 0 in a clinically relevant way, a 95% confidence interval was calculated.

## Results

### Intra-observer variability

The intra-individual deviations between the three measurements for the landmark-based analysis were determined for the different cleft types separately for the plaster cast and the intraoral scan. The determined mean values and medians are listed in Table 4.

**Table 4:** Mean intra-observer variability of landmark-based analysis in the different subtypes of CLP. SD = Standard deviation

object	Intra-observer variability (mean ± SD in mm)		
	CL / CP	UCLAP	BCLAP
**plaster cast**	0.28 ± 0.22	0.29 ± 0.21	0.36 ± 0.32
**Intraoral scan**	0.33 ± 0.28	0.30 ± 0.23	0.29 ± 0.21

The superimposition analysis revealed a total intra-observer variability of 0.09 ± 0.22 mm.

### Isolated cleft lip or cleft palate

**Fig. 2:**
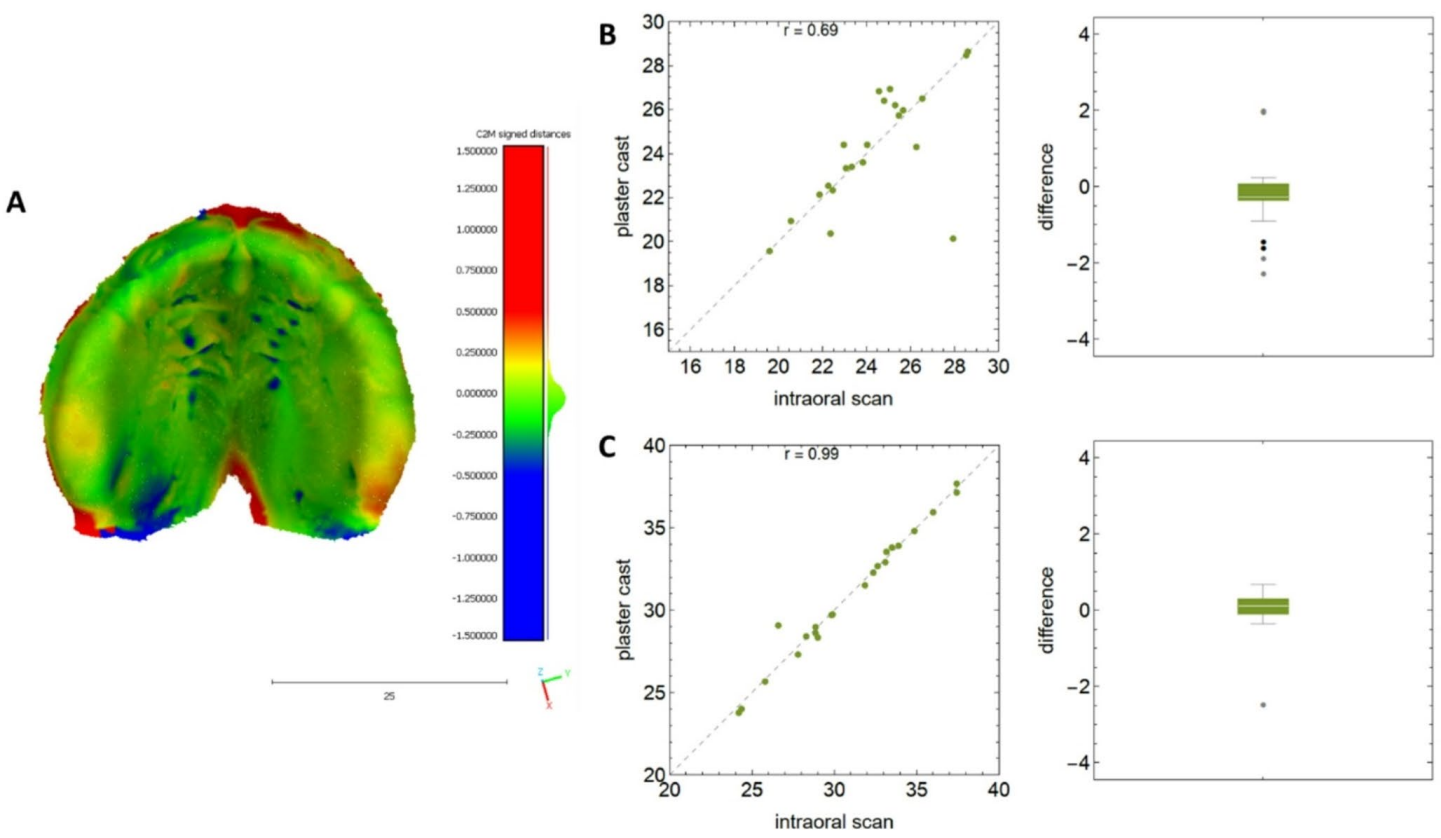
The accuracy between the plaster casts and intraoral scans in isolated CL and CP. (**A**) Representative colored illustration of the analyzed differences between– 1.5 mm (blue) and + 1.5 mm (red); (**B**) worst and (C) best correlation with corresponding boxplots of differences for this gap type

As shown in Table 5, all measured linear dimensions of the intraoral scans were, on average, 0.16 mm greater than the digitized plaster casts. The most significant deviation of 0.43 mm was observed for the length of the lateral alveolar segment on the right side. For the isolated cleft lip and palate, correlation coefficients between 0.69 (for the intercanine distance) and 0.99 (e.g. for the intertuberosity distance) were determined, indicating an agreement between the intraoral scans and the plaster models (Table 5; Fig. 2). The 3D superimposition of the digitized plaster casts and intraoral scans showed an excellent consistency between the two impression techniques. For this gap type, the RMS was − 0.07 ± 0.12 mm (mean ± SD) with a 95% confidence interval for the mean from − 0.16 to 0.02 mm.

### UCLAP

**Fig. 3:**
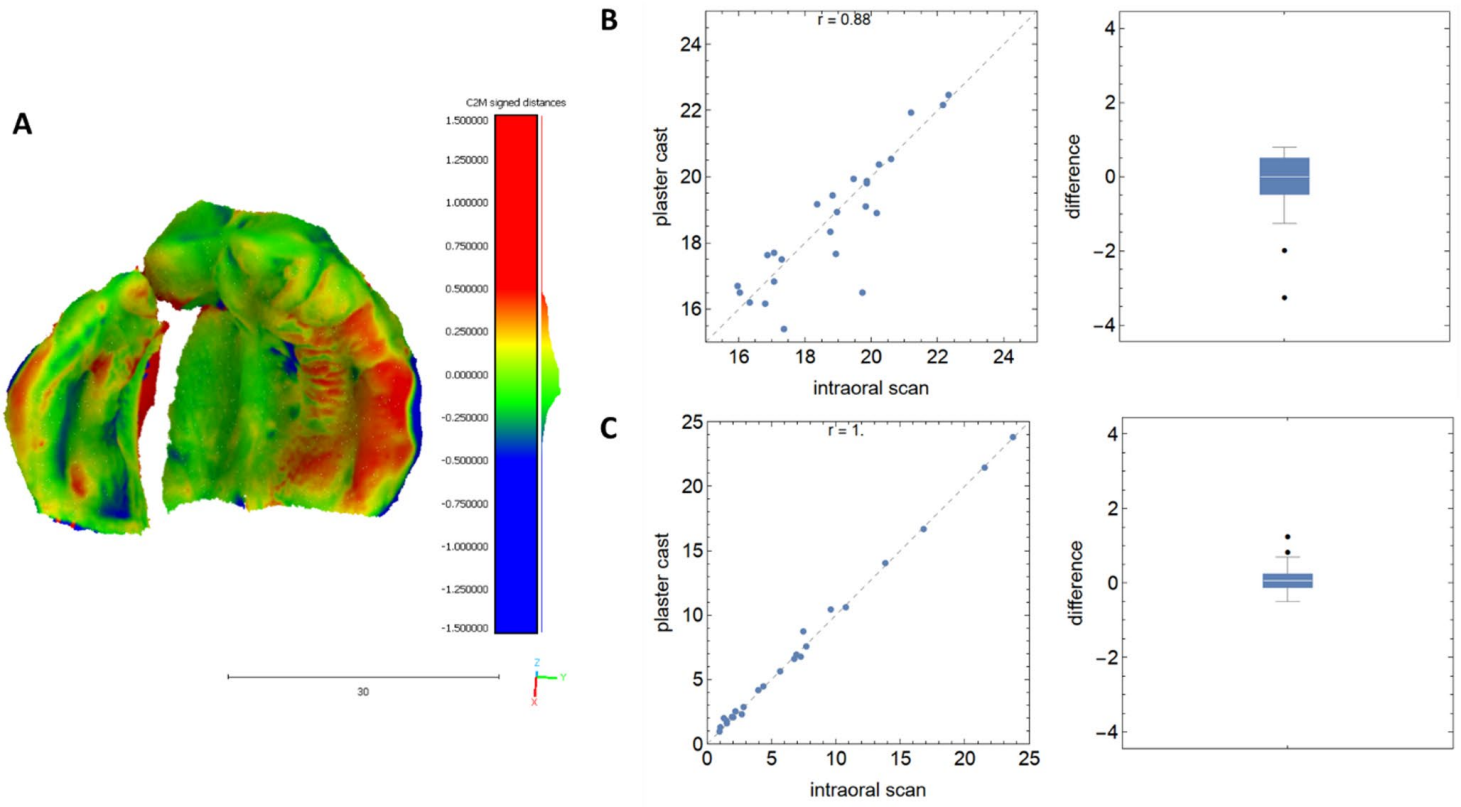
The accuracy between the plaster casts and intraoral scans in UCLAPs. (**A**) Representative colored illustration of the analyzed differences between– 1.5 mm (blue) and + 1.5 mm (red); (**B**) worst and (C) best correlation with corresponding boxplots of differences for this gap type

It was found that the plaster casts were also mostly larger than the intraoral scans in UCLAPs. The differences ranged from − 0.12 mm (cleft width) to -0.65 mm (canine distance). Surprisingly, two linear dimensions were smaller in the plaster models. These were the distance between the tuberosity points (T-T´) and the length of the alveolar processes on the cleft side (C-T, Table 5; Fig. 3). Nevertheless, the measured linear distances between the plaster model and intraoral scans correlate very well (Table 4; Fig. 3). The superimposition shows reduced areas of the alveolar segment on the cleft side when using the intraoral scan. These reductions occur mainly in deep-set, vertical areas. On the non-cleft side, however, enlarged dimensions of the alveolar segment can be detected with the help of the intraoral scan (Fig. 3). Based on the superimpositions, an RMS of -0.04 ± 0.18 mm (mean ± SD) was determined with a 95% confidence interval from − 0.17 to 0.09 mm.

### BCLAP

**Fig. 4:**
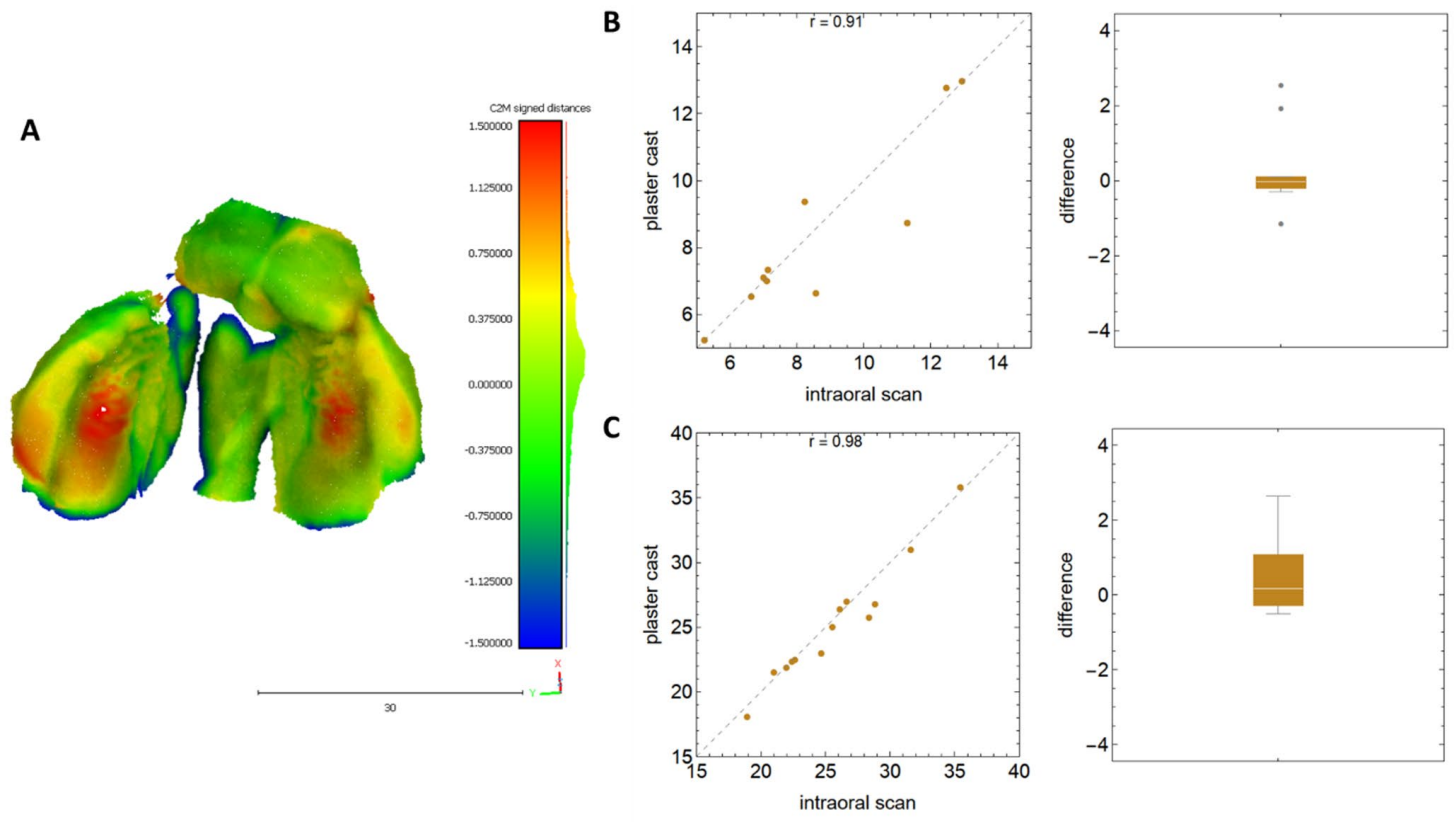
The accuracy between the plaster casts and intraoral scans in BCLAPs. (**A**) Representative colored illustration of the analyzed differences between– 1.5 mm (blue) and + 1.5 mm (red); (**B**) worst and (C) best correlation with corresponding boxplots of differences for this gap type

As already shown for the isolated CL / CP, the measured linear distances for BCLAP in the plaster model are on average smaller compared to the intraoral scans. However, it must be noted, with the exception of the cleft widths P1-P1´ and P2-P2´, all other measured distances show relatively large mean differences and standard deviations but with very good correlation (Table 5). The superimposition of a bilateral cleft shows a similar image to that of a unilateral cleft. Deep-seated structures of the cleft can either not be reproduced at all or only with considerably smaller dimensions using an intraoral scanner. However, in this cleft subtype, both alveolar segments are detected in an enlarged form compared to the alginate impression (Fig. 4). In these cases, the RMS was − 0.10 ± 0.17 mm (mean ± SD) with a 95% confidence interval from − 0.24 to 0.03 mm.

**Table 5:** Analyzing the mean difference (Δ) in maxillary arch linear dimensions in different subtypes of CLP. SD = Standard deviation

		intraoral scans versus plaster casts							
		T-T´	C´-T´	C-T	T-C´	C-T´	C-C´	P1-P1´	P2-P2´
**CL /CP**	Δ in mm (mean ± SD)	0.01 ± 0.61	0.43 ± 0.76	0.08 ± 0.89	0.20 ± 0.93	-0.13 ± 0.43	0.09 ± 1.99		
	r	0.99	0.97	0.95	0.95	0.99	0.69		
**UCLAP**	Δ in mm (mean ± SD)	0.12 ± 0.78	-0.14 ± 1.19	0.18 ± 0.95	-0.30 ± 0.89	-0.24 ± 1.26	-0.65 ± 1.32	-0.12 ± 0.38	
	r	0.98	0.94	0.88	0.96	0.95	0.92	1.0	
**BCLAP**	Δ in mm (mean ± SD)	0.34 ± 1.22	0.72 ± 1.0	0.58 ± 1.46	0.58 ± 1.37	0.89 ± 1.34	0.56 ± 1.05	-0.18 ± 0.92	0.29 ± 1.1
	r	0.96	0.95	0.92	0.94	0.95	0.98	0.92	0.91

## Discussion

Taking an impression is an essential part of dental treatment. One of the most recent advancements in dentistry, is the intraoral scanner, which enables the acquisition of highly precise digital impressions, transforming diagnostic and treatment processes. Digital impressions are the basis for digital diagnostics and treatment planning in orthodontics and restorative dentistry [12, 28–30]. The accuracy of these impressions therefore has a direct influence on the subsequent treatment steps. It is well known that for single-tooth restorations and fixed partial dentures, the accuracy of digital impressions is clinically satisfactory and similar to conventional impressions [11, 31]. Furthermore, the systematic review by Kong et al. found a 3D deviation between digital and alginate full-mouth impressions of only 0.09 mm, while Ahlholm and colleagues reported that the conventional method still provides the best results for full-mouth impressions [16, 17].

Some publications have shown that intraoral scanners can be used in infants with CLP [19–24]. In the context of developing digital workflows for the fabrication of presurgical orthopedic devices, there are several reports of individual to a few cases in infants with craniofacial disorders, including CLP [6, 9, 20–24, 32]. Regarding the comparison between digital and conventional impressions in infants with CLP, there are only a few case reports [22, 33, 34] but no detailed prospective studies. The present study evaluated the impression accuracy of digital impressions compared to conventional alginate impressions under clinical conditions for the fabrication of PSIOs. 52 infants from the Department of Oral and Maxillofacial Surgery at the University Hospital Carl Gustav Carus were available for the study. The current study included children with unilateral and bilateral CLAP and CP or CL. The aim of the study was not to investigate a subtype of CLP in more detail but to evaluate the accuracy of different impression methods for making PSIOs. Since PSIOs can be used for UCLAP, BCLAP and CP, it was necessary to include children with various subtypes of CLP in this study. Regardless of the form of CLP, the intraoral scans and the plaster casts correlated very well with each other. The measured linear dimensions of the intraoral scans were greater than those of the digitized plaster casts. The smallest mean differences in linear dimensions and the best superimposition results were found for isolated CL/CP with values between 0.01 and 0.43 mm, followed by UCLAPs (-0.12– -0.65 mm mean difference). The biggest differences in these two groups occur on the one hand on the left side and on the other hand in the intercanine distance. Our linear measurements are consistent with those found in other studies. When using linear measurement methods, a total error of between 0.23 mm and 1.33 mm was found in 10 infants with UCLAP, depending on where the measurement was taken. When measuring the distance between the canines, the greatest variation was found [35]. The quality of the intraoral scans and plaster models played a very important role in the landmark-based analysis of the accuracy between the two impression methods. This is particularly noticeable in the fact that the reference points for the linear measurements were difficult to identify when the impressions were of poor quality.

In the meantime, however, it has been shown that the landmark-based analysis of models is not sufficiently meaningful, especially not for the three-dimensional changes in cleft palate. Rather, superimposition should be used to analyze the accuracy between the intraoral scan and the plaster cast in these cases. We were able to show clearly that linear measurements can give false information about the accuracy of the two impression methods. Measuring only individual distances gives the impression that the accuracy of the impressions taken with the intraoral scans is not consistent in the cleft patients. This is particularly evident with BCLAP. In the impressions of 13 children with BCLAP that we examined, the mean deviation between the two impression methods in the landmark-based analysis was between 0.19 and 0.89 mm. A difference of > 0.3 mm was defined as clinically relevant. Therefore, some of the differences measured in the 2D analysis would be considered clinically relevant. However, this is not the case. The 3D analysis showed a mean deviation of -0.18 mm for the same impressions, which is not clinically relevant. Even for the isolated and unilateral gaps, the 3D analysis was able to detect mean deviations between − 0.04 and − 0.07 mm between the intraoral scans and digitized plaster models. This agrees with previous publications. In the study by ElNaghy et al. on two infants with UCLAP, it was shown that the intraoral scan with the Trios 3Shape scanner showed excellent agreement with the conventional impression. When digitally superimposing the scans, only minimal differences between 0.01 and 0.1 mm were found [34]. Dalessandri et al. found no significant differences in the accuracy of plate fabrication based on virtual digital models compared to conventional plaster casts in one patient with bilateral and two patients with unilateral CLP [33]. Meanwhile, there are also individual case studies that show a complete digital workflow for the production of PSIOs [20, 21]. According to these, intraoral scans are suitable for the production of these special orthodontic appliances. The differences between conventional and digital impression methods are not clinically relevant. In most cases, it can be assumed that the accuracy and the quality of the intraoral scans can be considered better. Due to the fact that the actual impression material displaces the mucous membrane in the mouth (resilience) and that there is a further shrinkage during the production of the plaster models, the intraoral scans must actually be somewhat larger. This could be clearly demonstrated in this study. The sometimes large differences between the alginate impression and the intraoral scan can initially be traced back to the impression trays. Customized impression trays are not available for CLP newborns, nor are they commercially available. Furthermore, the existing impression trays are used regardless of the cleft type. This explains why the impressions for isolated cleft lip and palate show the best results, whereas the fit of the impression trays is less accurate for BCLAPs. Very often, a dislocated and highly mobile premaxillary segment occurs in bilateral clefts due to anatomical conditions. Lateral segments are also often severely displaced in a vertical direction in cleft patients. These conditions can cause difficulties when creating the intraoral scan.

The main difficulties with intraoral scanning are the size of the scanner head and the scanning software [15]. There are no intraoral scanners on the market designed specifically for children and neonates. All intraoral scanners are designed for adults with correspondingly large scanning heads. Furthermore, the current trend is to increase the size of the scanner head rather than reduce it in order to increase the speed of the scan. It is therefore understandable that it is sometimes very difficult to insert the large scanner heads into the small mouths of newborns and obtain accurate intraoral scans. It is well known that the limited mouth opening, visibility and presence of orogastric tubes in infants with clefts can often complicate impression taking. In our study, we found that the quality of intraoral scans improved with increasing patient age. To support this claim, the quality of the impressions of eight children who had an intraoral scan and a plaster model made at 4–5 months and 9–12 months of age was evaluated (data not shown). It is known that the existing scanner software is designed to capture complete dental arches and dentitions in adults. For this reason, the discontinuous alveolus and the cleft palate may be interpreted as redundant details and surfaces. The depth of field of the scanner is also a problem when deeper structures are to be imaged and a “virtual bridge” is to be built between the cleft segments. These difficulties have also been described in other studies [20, 22, 23, 34]. Recently, a good correlation between digital and conventional impressions in the maxilla and mandible has been demonstrated using a TRIOS scanner, even in edentulous adult patients [18] and mixed dentition [14].

In any case, it must be pointed out that an intraoral scan in newborns and infants should only be performed by trained personnel. Training, repetition and a standardized scanning protocol can achieve a certain level of professionalism and reproducibility, as has already been demonstrated for intraoral scans of adults [20, 36].

## Conclusion

The null hypothesis established at the beginning was proven. There is no clinically relevant difference between the intraoral scan and the plaster casts, regardless of the cleft type. This study confirms that intraoral scanning is a reliable and clinical valuable alternative to conventional alginate impressions for infants with CLP. Intraoral scanning eliminates risks associated with traditional methods, providing a safer and more efficient workflow for PSIO. When investigating the accuracy of an applied impression method, 3D analysis should always be preferred to 2D analysis as it can provide more accurate information. Despite minor differences in dimensional accuracy, these variations are clinically negligible and do not impact treatment planning or appliance fabrication. Further advancements in scanner technology and standardized protocols will essential fully integrate this method in the everyday cleft care.

## Data Availability

No datasets were generated or analysed during the current study.

## References

[1] Mossey PA, Little J, Munger RG, Dixon MJ, Shaw WC (2009) Cleft lip and palate. Lancet 374:1773–1785. 10.1016/S0140-6736(09)60695-4 19747722 10.1016/S0140-6736(09)60695-4

[2] Tolarova MM, Cervenka J (1998) Classification and birth prevalence of orofacial clefts. Am J Med Genet 75:126–137 9450872

[3] Voigt A, Radlanski RJ, Sarioglu N, Schmidt G (2017) [Cleft lip and palate]. Pathologe 38:241–247. 10.1007/s00292-017-0313-x 28653248 10.1007/s00292-017-0313-x

[4] McNeil CK (1950) Orthodontic procedures in the treatment of congenital cleft palate. Dent Rec (London) 70:126–132 24537837

[5] Prahl C, Kuijpers-Jagtman AM, Van ‘t Hof MA, Prahl-Andersen B (2003) A randomized prospective clinical trial of the effect of infant orthopedics in unilateral cleft lip and palate: prevention of collapse of the alveolar segments (Dutchcleft). Cleft Palate Craniofac J 40:337–342. 10.1597/1545-1569_2003_040_0337_arpcto_2.0.co_2 12846598 10.1597/1545-1569_2003_040_0337_arpcto_2.0.co_2

[6] Zarean P, Zarean P, Thieringer FM, Mueller AA, Kressmann S, Erismann M, Sharma N, Benitez BK (2022) A Point-of-Care digital workflow for 3D printed passive presurgical orthopedic plates in cleft care. Child (Basel) 9. 10.3390/children9081261 10.3390/children9081261 PMC9406563 36010151

[7] Chate RA (1995) A report on the hazards encountered when taking neonatal cleft palate impressions (1983–1992). Br J Orthod 22:299–307. 10.1179/bjo.22.4.299 8580095 10.1179/bjo.22.4.299

[8] Reichert F, Amrhein P, Uhlemann F (2017) Unnoticed aspiration of palate plate impression material in a neonate: diagnosis, therapy, outcome. Pediatr Pulmonol 52:E58–E60. 10.1002/ppul.23710 28440911 10.1002/ppul.23710

[9] Xepapadeas AB, Weise C, Frank K, Spintzyk S, Poets CF, Wiechers C, Arand J, Koos B (2020) Technical note on introducing a digital workflow for newborns with craniofacial anomalies based on intraoral scans - part I: 3D printed and milled palatal stimulation plate for trisomy 21. BMC Oral Health 20:20. 10.1186/s12903-020-1001-4 31973720 10.1186/s12903-020-1001-4 PMC6979345

[10] Ferreira JB, Christovam IO, Alencar DS, da Motta AFJ, Mattos CT, Cury-Saramago A (2017) Accuracy and reproducibility of dental measurements on tomographic digital models: a systematic review and meta-analysis. Dentomaxillofac Radiol 46:20160455. 10.1259/dmfr.20160455 28362170 10.1259/dmfr.20160455 PMC5988188

[11] Mangano F, Gandolfi A, Luongo G, Logozzo S (2017) Intraoral scanners in dentistry: a review of the current literature. BMC Oral Health 17:149. 10.1186/s12903-017-0442-x 29233132 10.1186/s12903-017-0442-x PMC5727697

[12] Peluso MJ, Josell SD, Levine SW, Lorei BJ (2004) Digital models: an introduction. Semin Orthod 10:226–238

[13] Kravitz ND, Groth C, Jones PE, Graham JW, Redmond WR (2014) Intraoral digital scanners. J Clin Orthod 48:337–347 25083754

[14] Liczmanski K, Stamm T, Sauerland C, Blanck-Lubarsch M (2020) Accuracy of intraoral scans in the mixed dentition: a prospective non-randomized comparative clinical trial. Head Face Med 16:11. 10.1186/s13005-020-00222-6 32430023 10.1186/s13005-020-00222-6 PMC7236363

[15] Aswani K, Wankhade S, Khalikar A, Deogade S (2020) Accuracy of an intraoral digital impression: A review. J Indian Prosthodont Soc 20:27–37. 10.4103/jips.jips_327_19 32089596 10.4103/jips.jips_327_19 PMC7008627

[16] Ahlholm P, Sipila K, Vallittu P, Jakonen M, Kotiranta U (2018) Digital versus conventional impressions in fixed prosthodontics: A review. J Prosthodont 27:35–41. 10.1111/jopr.12527 27483210 10.1111/jopr.12527

[17] Kong L, Li Y, Liu Z (2022) Digital versus conventional full-arch impressions in linear and 3D accuracy: a systematic review and meta-analysis of in vivo studies. Clin Oral Investig 26:5625–5642. 10.1007/s00784-022-04607-6 35786783 10.1007/s00784-022-04607-6

[18] Papaspyridakos P, Vazouras K, Chen YW, Kotina E, Natto Z, Kang K, Chochlidakis K (2020) Digital vs conventional implant impressions: A systematic review and Meta-Analysis. J Prosthodont 29:660–678. 10.1111/jopr.13211 32613641 10.1111/jopr.13211

[19] Chalmers EV, McIntyre GT, Wang W, Gillgrass T, Martin CB, Mossey PA (2016) Intraoral 3D scanning or dental impressions for the assessment of dental arch relationships in cleft care: which is superior?? Cleft Palate Craniofac J 53:568–577. 10.1597/15-036 26623548 10.1597/15-036

[20] Gong X, Dang R, Xu T, Yu Q, Zheng J (2020) Full digital workflow of Nasoalveolar molding treatment in infants with cleft lip and palate. J Craniofac Surg 31:367–371. 10.1097/SCS.0000000000006258 32049908 10.1097/SCS.0000000000006258

[21] Krey KF, Ratzmann A, Metelmann PH, Hartmann M, Ruge S, Kordass B (2018) Fully digital workflow for presurgical orthodontic plate in cleft lip and palate patients. Int J Comput Dent 21:251–259 30264054

[22] Patel J, Winters J, Walters M (2019) Intraoral digital impression technique for a neonate with bilateral cleft lip and palate. Cleft Palate Craniofac J 56:1120–1123. 10.1177/1055665619835082 30857398 10.1177/1055665619835082

[23] Weise C, Frank K, Wiechers C, Weise H, Reinert S, Koos B, Xepapadeas AB (2022) Intraoral scanning of neonates and infants with craniofacial disorders: feasibility, scanning duration, and clinical experience. Eur J Orthod 44:279–286. 10.1093/ejo/cjab068 34532733 10.1093/ejo/cjab068

[24] Carter CB, Gallardo FF Jr., Colburn HE, Schlieder DW (2022) Novel digital workflow for Nasoalveolar molding and postoperative nasal stent for infants with cleft lip and palate. Cleft Palate Craniofac J 10556656221095393. 10.1177/10556656221095393 10.1177/10556656221095393 35477311

[25] Jeong YG, Lee WS, Lee KB (2018) Accuracy evaluation of dental models manufactured by CAD/CAM milling method and 3D printing method. J Adv Prosthodont 10:245–251. 10.4047/jap.2018.10.3.245 29930795 10.4047/jap.2018.10.3.245 PMC6004348

[26] Aretxabaleta M, Unkovskiy A, Koos B, Spintzyk S, Xepapadeas AB (2021) Accuracy evaluation of additively and subtractively fabricated palatal plate orthodontic appliances for newborns and infants-An in vitro study. Mater (Basel) 14. 10.3390/ma14154103 10.3390/ma14154103 PMC8347028 34361299

[27] Oday R, Abid M, Dziedzic A (2024) The accuracy and retention of presurgical infant orthopaedics constructed from different polymer materials: A comparative study. J Taibah Univ Med Sci 19:379–389. 10.1016/j.jtumed.2024.01.005 38370166 10.1016/j.jtumed.2024.01.005 PMC10874750

[28] Fleming PS, Marinho V, Johal A (2011) Orthodontic measurements on digital study models compared with plaster models: a systematic review. Orthod Craniofac Res 14:1–16. 10.1111/j.1601-6343.2010.01503.x 21205164 10.1111/j.1601-6343.2010.01503.x

[29] Rheude B, Sadowsky PL, Ferriera A, Jacobson A (2005) An evaluation of the use of digital study models in orthodontic diagnosis and treatment planning. Angle Orthod 75:300–304. 10.1043/0003-3219(2005)75[300:AEOTUO]2.0.CO;2 15898364 10.1043/0003-3219(2005)75[300:AEOTUO]2.0.CO;2

[30] Zhang F, Suh KJ, Lee KM (2016) Validity of intraoral scans compared with plaster models: an In-Vivo comparison of dental measurements and 3D surface analysis. PLoS ONE 11:e0157713. 10.1371/journal.pone.0157713 27304976 10.1371/journal.pone.0157713 PMC4909173

[31] Ye J, Wang S, Wang Z, Liu Y, Sun Y, Ye H, Zhou Y (2023) Comparison of the dimensional and morphological accuracy of three-dimensional digital dental casts digitized using different methods. Odontology 111:165–171. 10.1007/s10266-022-00736-2 36068382 10.1007/s10266-022-00736-2

[32] Xepapadeas AB, Weise C, Frank K, Spintzyk S, Poets CF, Wiechers C, Arand J, Koos B (2020) Technical note on introducing a digital workflow for newborns with craniofacial anomalies based on intraoral scans - part II: 3D printed Tubingen palatal plate prototype for newborns with robin sequence. BMC Oral Health 20:171. 10.1186/s12903-020-01159-7 32546229 10.1186/s12903-020-01159-7 PMC7298740

[33] Dalessandri D, Tonni I, Laffranchi L, Migliorati M, Isola G, Bonetti S, Visconti L, Paganelli C (2019) Evaluation of a digital protocol for Pre-Surgical orthopedic treatment of cleft lip and palate in newborn patients: A pilot study. Dent J (Basel) 7. 10.3390/dj7040111 10.3390/dj7040111 PMC6960660 31835442

[34] ElNaghy R, Amin SA, Hasanin M (2022) Evaluating the accuracy of intraoral direct digital impressions in 2 infants with unilateral cleft lip and palate compared with digitized conventional impression. Am J Orthod Dentofac Orthop 162:403–409. 10.1016/j.ajodo.2021.09.015 10.1016/j.ajodo.2021.09.015 36049869

[35] Braumann B, Keilig L, Bourauel C, Jager A (2002) Three-dimensional analysis of morphological changes in the maxilla of patients with cleft lip and palate. Cleft Palate Craniofac J 39:1–11. 10.1597/1545-1569_2002_039_0001_tdaomc_2.0.co_2 11772163 10.1597/1545-1569_2002_039_0001_tdaomc_2.0.co_2

[36] Muller P, Ender A, Joda T, Katsoulis J (2016) Impact of digital intraoral scan strategies on the impression accuracy using the TRIOS pod scanner. Quintessence Int 47:343–349. 10.3290/j.qi.a35524 26824085 10.3290/j.qi.a35524

